# Investigation of PBT-AP Interactions in PBT-Based Solid Propellants: A Combined Density Functional Theory and Molecular Dynamics Study

**DOI:** 10.3390/polym17111492

**Published:** 2025-05-27

**Authors:** Kun Liu, Xinlu Cheng

**Affiliations:** Institute of Atomic and Molecular Physics, Sichuan University, Chengdu 610065, China; 2022226060019@stu.scu.edu.cn

**Keywords:** DFT, BAMO-THF copolymer, ammonium perchlorate, molecular dynamics simulations

## Abstract

Poly(3,3-bis(azidomethyl)oxetane(BAMO)-tetrahydrofuran(THF)) copolymer (PBT) and ammonium perchlorate (AP) are critical components of solid rocket propellants, where their interfacial bonding mechanisms and temperature-dependent mechanical properties are pivotal to propellant reliability. In this study, density functional theory (DFT) calculations were employed to evaluate the adsorption energies between common AP crystal surfaces and PBT units, identifying the most energetically favorable adsorption configurations. The atomic configurations and charge transfer characteristics at the PBT-AP interface were systematically analyzed. Molecular dynamics (MD) simulations were further conducted to determine the thermally stable operating range of the PBT-AP system. The results reveal a strong temperature dependence of mechanical performance, with viscous failure mechanisms and damage thresholds during static tensile processes investigated across varying temperatures. Notably, mechanical properties remain stable below 60 °C but deteriorate significantly above this temperature. This study elucidates the influence of a PBT-AP interfacial microstructure and temperature on mechanical performance and tensile fracture damage boundaries, providing crucial insights for the design, formulation, and safe application of PBT-based solid rocket propellants.

## 1. Introduction

Solid propellants, referring to solid energetic materials that provide power for the propulsion systems of missiles, spacecraft, and other vehicles, play an irreplaceable role in military and aerospace applications [1,2]. Research on solid propellants is important for both defensive and civil applications. The rapid advancement of solid propellant technology primarily occurred in the decades following World War II, during which substantial resources were invested globally to achieve breakthroughs. This period witnessed the emergence of numerous novel energetic materials, such as polysulfide (PS), carboxyl-terminated polybutadiene (CTPB), and hydroxyl-terminated polybutadiene (HTPB) propellants, significantly enhancing rocket range and velocity.

As the application scenarios for rockets diversified—including naval, vehicular, and airborne platforms—design requirements have expanded beyond high energy density to prioritize safety [3,4]. For instance, the widely used HTPB propellant poses risks due to violent explosions under thermal stimulation, threatening military equipment and personnel. To address these challenges, insensitive propellants, such as HTPE-based and PBT(Poly(3,3-bis(azidomethyl)oxetane(BAMO)-tetrahydrofuran(THF))copolymer)-based formulations, have been developed over years of research and are now approaching practical application. Among these, PBT-based propellants, containing azide groups, exhibit superior energy output and are internationally recognized as highly promising solid propellants. Nevertheless, the critical aspects of PBT propellants—including formulation design, component optimization, and physicochemical mechanisms—remain underexplored. Thus, foundational studies on PBT propellant composition and component interactions are essential. Such research not only validates existing experimental and theoretical findings but also accumulates data to support the future development of diverse propellants, leveraging big data and machine learning to identify advanced energetic materials with reduced resource expenditure.

Current research on PBT-based solid propellants is predominantly experimental. For example, Toshio et al. [5] elucidated the thermochemical decomposition pathways of PBAMO, BAMO/THF (60:40) copolyether, and cross-linked BAMO/THF systems using a combined DTA-TG-IR analysis system and proposed a thermal decomposition mechanism for BAMO/THF. Their study revealed a biphasic decomposition mechanism: (1) the rapid exothermic cleavage of azide (-N3) groups, resulting in a 35% mass loss through nitrogen gas (N_2_) evolution and forming imine (-CH=NH) intermediates, as evidenced by infrared spectral shifts (2100 → 1650/3400 cm^−1^); (2) the gradual degradation of the carbon backbone at temperatures above 520 K, yielding residual char (65% mass loss). Cross-linked systems demonstrated analogous N3 decomposition kinetics but exhibited significantly suppressed gas-phase reactivity due to stabilization by the network structure; Chen et al. [6] experimentally studied the relationship between aging and temperature in PBT-based solid propellants. The research results indicate that the thermal oxidation process of PBT elastomers involves multiple coupled mechanisms: the initial stage is dominated by hydrogen bond cleavage, followed by the intensified degradation of polyurethane segments, and finally, the oxidation cross-linking of the polyether backbone becomes the predominant reaction; Xie et al. [7] evaluated the effects of multiple curing agents, including HDI, TDI, IPDI, and N-100, on tensile strength and elongation properties. The study revealed that the multifunctional curing agent N-100 significantly enhanced the tensile strength of the propellant (reaching 0.734 MPa at 25 °C and 0.656 MPa at 50 °C) due to its high cross-linking density. However, this increased rigidity concurrently led to a marked reduction in elongation (21.2–29.9% across different temperatures). In contrast, the difunctional curing agent TDI achieved a balanced performance—improving strength while maintaining superior elongation (50.3–54.7%)—which was attributed to its moderate cross-linking that preserved the flexibility of the network structure.

In computational simulations, limited studies exist. For instance, Jia et al. [8] investigated the synergistic mechanisms between the cross-linking degree of the PBT matrix and ammonium perchlorate (AP) surface defects on the interfacial adsorption behavior of propellants; Chen et al. [9] investigated the structure–property relationships of the model compounds of BAMO-THF copolyether and the curing agent using density functional theory. By calculating and analyzing electrostatic potential maps, they explored potential reaction sites involved in the curing process. The study revealed multiple reactive sites between TDI and PBT. Furthermore, the thermodynamically stable structures during the curing reaction were identified, and the transition states in the reaction pathways were calculated, enabling the determination of the most probable curing reaction pathway and its corresponding activation Gibbs free energy. This work holds significant scientific importance for gaining in-depth insights into the interaction mechanisms between the PBT binder and TDI curing agent, as well as elucidating the fundamental principles governing the curing reaction mechanism.

However, computational studies on PBT-based solid propellants remain scarce, particularly lacking in-depth analyses of charge distribution, bonding interactions, and mechanical properties at the atomic/molecular level. Therefore, this study will conduct a detailed investigation at the atomic interaction level using density functional theory (DFT) calculations to thoroughly explore the interactions between atoms in the commonly used oxidizer ammonium perchlorate (AP) and PBT molecules within propellants. Meanwhile, molecular dynamics (MD) simulations offer robust parameter control and precision, serving as reliable tools for investigating material structures and properties [10,11,12,13]. Given the challenges and safety risks associated with experimental studies of high-energy-density materials under extreme conditions (e.g., high temperature and pressure), this study employs MD simulations to evaluate the mechanical behavior of the PBT-AP interface.

## 2. Theoretical Models and Computational Methods

### 2.1. Model Construction

**Figure 1:**
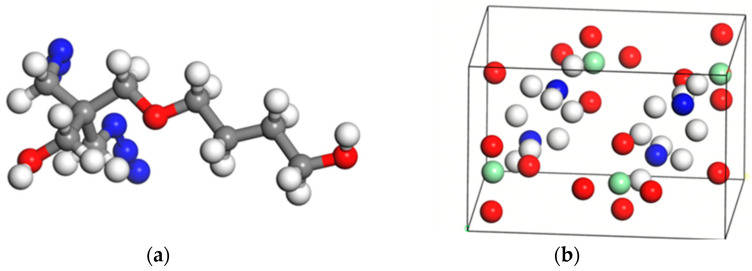
(**a**) PBT structural unit; (**b**) AP initial crystal cell model.

DFT calculations were performed using the Vienna Ab initio Simulation Package (VASP 5.4.4) [14,15]. Given the structural complexity of the BAMO-THF (PBT) copolymer, DFT calculations focused on its repeating unit as the representative model. Initially, the fundamental structural unit of the PBT binder was built within the Materials Studio 2020 (MS) software. The geometric optimization of the unit was conducted using the CASTEP module, followed by energy minimization via the DMol3 module. The final optimized stable configuration is illustrated in Figure 1.

In this study, the ammonium perchlorate crystal structure (NH_4_ClO_4_) was obtained from the American Mineralogist Crystal Structure Database, with the space group *Pnma*. Hydrogen atoms were added to complete the atomic coordinates, followed by the geometric optimization of the crystal cell. The optimized lattice parameters were determined as a = 6.01 Å, b = 7.37 Å, and c = 9.06 Å, with angles *α* = *β* = *γ* = 90°, yielding a calculated density of 1.95 g/cm^3^.

Numerous studies have investigated the crystal surfaces of ammonium perchlorate. For instance, Khan et al. [16] employed DFT to evaluate the stability of AP surfaces in an ethylene glycol solvent environment, ranking them as (210) > (001) > (101) > (011) > (100). In contrast, Yeh et al. [17] reported differing results using MD simulations, identifying (210), (001), and (010) as the most stable surfaces. Experimental work by Kang et al. further demonstrated that (210) and (001) are the predominant cleavage planes during AP crystal deformation. Synthesizing these findings, the (001), (210), (011), and (201) surfaces—exhibiting relatively high stability—were selected for interfacial modeling in this study.

**Figure 2:**
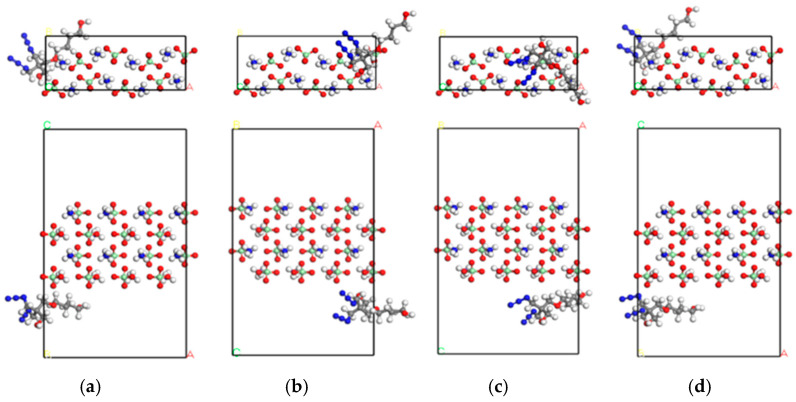
(**a**–**d**) Front and top views of four adsorption configurations of PBT structural units on the (001) surface of the AP crystal.

To mitigate computational complexity in VASP simulations for multi-atomic systems, a 2 × 1 × 2 supercell was constructed. To ensure computational accuracy and efficiency, multiple supercell configurations (2 × 1 × 1, 2 × 1 × 2, 2 × 2 × 2, 3 × 3 × 3) were tested. The 2 × 1 × 2 model was ultimately adopted as it sufficiently captures interfacial charge redistribution while minimizing computational resource demands. The Adsorption Locator module in MS was then utilized to dock the PBT structural unit onto each surface, yielding distinct adsorption configurations. Preliminary calculations revealed that the (001) surface exhibited the lowest adsorption energy, prompting a further analysis of its four most energetically favorable configurations (Figure 2). In the figure, atomic species are color-coded as follows: nitrogen (blue), hydrogen (white), carbon (gray), oxygen (red), and chlorine (green).

**Figure 3:**
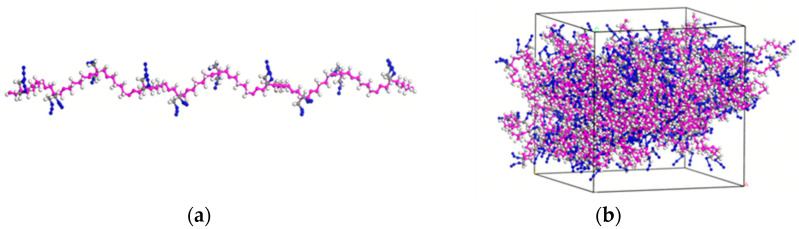
(**a**) PBT polymer chain; (**b**) PBT polymer simulation box.

For MD simulations, a polymerized PBT chain with a degree of polymerization (DP) of 10 was generated in MS. Fifty such chains were packed into a simulation box with a target density of 1.6 g/cm^3^. After geometric optimization to eliminate atomic overlaps and high-energy regions, the equilibrated system (Figure 3b) was obtained for subsequent mechanical analyses.

**Figure 4:**
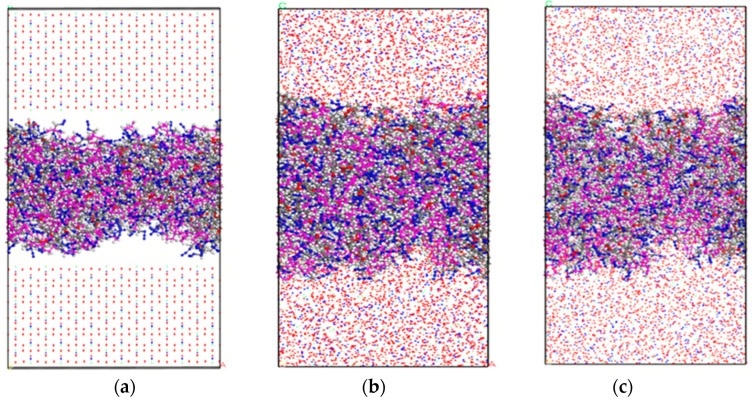
(**a**) Layered structure after geometric optimization; (**b**) NVT relaxation result; (**c**) NPT relaxation result.

A periodic AP supercell with dimensions of 7 × 6 × 3 was constructed, and the (001) surface was cleaved from the optimized crystal. The layered structure was then assembled by integrating the PBT polymer simulation box (Figure 3b) with the AP surface. The geometric optimization of the layered model yielded the initial equilibrated structure shown in Figure 4a.

Subsequently, an NVT ensemble relaxation was performed at a temperature of 298 K. Since the relaxation time of the PBT chain in the NVT ensemble at 298 K was 4.85 ps, the time step was set to 0.05 fs for 200,000 steps (total simulation time: 100 ps), resulting in the intermediate configuration depicted in Figure 4b. This was followed by an NPT ensemble relaxation under identical temperature (298 K) and a pressure of 1.0 GPa, maintaining the same time step and simulation duration (100 ps), which produced the final equilibrated structure (Figure 4c). All subsequent molecular dynamics simulations were conducted using Figure 4c configuration as the initial state.

### 2.2. Selection of Computational Methods

For the computational methodology, structural optimization and DFT calculations were performed on the four configurations in Figure 2 using the VASP. The electron–ion interactions were described by the Projected Augmented Wave (PAW) method, while the exchange–correlation potential was approximated by the Perdew–Burke–Ernzerhof (PBE) functional under the generalized gradient approximation (GGA) to optimize the geometry and obtain the initial electronic structure. Previous studies have demonstrated that van der Waals (vdW) forces play a critical role in such systems [18,19]. Currently, methods capable of handling long-range weak interactions include DFT-D2/D3 corrections, van der Waals density functional (vdW-DF) [20], and range-separated (RS) functionals [21]. Therefore, the DFT-D3 method with Becke–Jonson damping was employed in all DFT calculations to account for these interactions [22].

Simulated annealing, widely employed for studying structural stability [23,24,25] and energy minimization [25,26,27], was adopted in this study to analyze configuration (a) in Table 1. The calculations were conducted using the MD module within the VASP, with identical pseudopotential files (POTCAR) and parameters as the preceding adsorption energy calculations. Critical input parameters were configured as follows: plane-wave cutoff energy (ENCUT): 400 eV; electronic energy convergence criterion (EDIFF): 1 × 10^−4^ eV; time step (POTIM): 1.0 fs; temperature control: Nose–Hoover thermostat (SMASS = −3).

MD simulations for mechanical properties and tensile behavior were performed in MS using the COMPASSII force field, which integrates quantum mechanical calculations and experimental data to provide widely validated parameters. This force field has been successfully applied to study high-energy-density materials and polymers [28,29,30,31,32]. The Mechanical Properties function in the Forcite module was employed with the following settings: the method was constant strain, Forcefield was COMPASS II, charges was Forcefield assigned, cutoff distance = 12.5 Å, buffer width = 2 Å, number of steps for each strain = 5, and maximum strain amplitude = 0.003. The tensile simulations were automated via Perl scripts with uniaxial tension along the Z-axis under applied pressures of 0.5, 1, 2, and 3 GPa. Forcefield was COMPASS II and the direction of stress = ZZ, and the system underwent five pre-equilibration cycles, with each cycle consisting of 50,000 steps. After each stress application, the system was equilibrated for 50,000 steps in the NPT ensemble, and sampling was performed every 50,000 steps.

## 3. Results and Data Analysis

### 3.1. DFT Calculations

Previous studies on PBT-based propellants have primarily focused on experimental performance evaluations, with limited theoretical insights into their interaction mechanisms. To address this gap, we employed DFT to systematically investigate the atomic-level interactions between the PBT unit and AP crystals, aiming to elucidate the fundamental principles governing their interfacial behavior.

#### 3.1.1. DFT Calculations and Adsorption Energy Analysis

We performed calculations on the PBT structural unit, AP crystal surfaces, and the four adsorption configurations in Figure 2 using the VASP. The adsorption energy was subsequently calculated using Equation (1):

E_ab_ = E_total_ − E_clean_ − E_suf_ (1)

Here, E_ab_ represents the adsorption energy between the PBT unit and the AP system, E_total_ denotes the total energy of the combined system, E_clean_ is the energy of the pristine AP surface, and E_suf_ corresponds to the energy of the PBT unit prior to adsorption. The calculated results are summarized in Table 1.

A comparison of the calculated results with prior studies reveals significantly higher adsorption energies, indicating a lower total system energy and enhanced thermodynamic stability. These findings provide a robust theoretical explanation for the desensitization effect observed in PBT-based propellants. Furthermore, the post-calculation atomic configuration analysis (e.g., Figure 2) demonstrates that the PBT unit remains firmly adsorbed on the AP crystal surface without dissociation, further corroborating the stability of the interfacial structure.

#### 3.1.2. Charge Density Analysis

**Figure 5:**
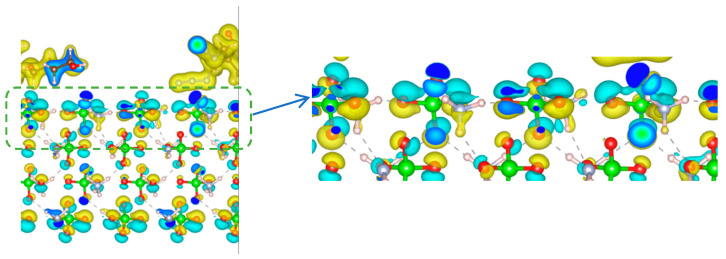
Charge density difference (CDD) plots of the PBT unit and AP crystal.

To further investigate configuration (a), we conducted a detailed charge density analysis. First, the AP crystal surface and PBT unit in configuration (a) were isolated, and all atomic positions were fixed. Subsequent optimization calculations were performed using VASP to obtain the charge densities of the PBT unit, AP surface, and configuration (a) individually. The charge density difference (CDD) was then derived by subtracting the charge densities of the isolated PBT unit and AP surface from that of the combined PBT-AP system using VESTA. The resulting CDD plot is shown in Figure 5.

In Figure 5, the left figure shows the original differential charge density map, while the right figure provides a magnified view of the PBT unit binding to the AP crystal plane. In the figure, regions with increased electron density are represented in yellow, while areas with decreased electron density are depicted in blue. From Figure 5, it can be clearly observed that for oxygen atoms (red) on the AP crystal plane, the electron density decreases on the side adjacent to the PBT unit and increases on the side facing the crystal interior. This indicates significant electrostatic repulsion between the negatively charged azide groups of the PBT unit and the negatively charged oxygen ions in the AP crystal during their adsorption process. When analyzing the charge density changes of nitrogen atoms, a similar trend to oxygen atoms is observed. This occurs because nitrogen atoms in the AP crystal belong to the NH_4_^+^ group and also carry negative electronegativity, leading to comparable repulsion with the negative charges in the azide groups.

An analysis of hydrogen atom charge variations reveals that H atoms near the PBT unit adsorption sites exhibit shifted charge density toward the PBT unit, suggesting electrostatic attraction. In contrast, distant H atoms show no charge density changes. This demonstrates that during PBT-AP adsorption, short-range H atoms enhance adsorption performance through electrostatic attraction, while long-range H atoms exhibit negligible electrostatic interactions with the PBT unit.

The above analysis shows that the repulsive forces manifested by charge variations in O and N atoms dominate over the relatively weak short-range electrostatic attraction from H atoms. Consequently, the overall electrostatic interaction between the PBT unit and AP crystal is net repulsive. For PBT binder adhesion performance, such electrostatic repulsion would negatively impact bonding effectiveness. To optimize PBT-based solid propellants, formulation adjustments could include adding components to suppress this electrostatic repulsion or modifying the PBT unit to reduce the electronegativity of its azide groups, thereby enhancing adhesive performance.

#### 3.1.3. Atomic Distance and Bonding Analysis

**Figure 6:**
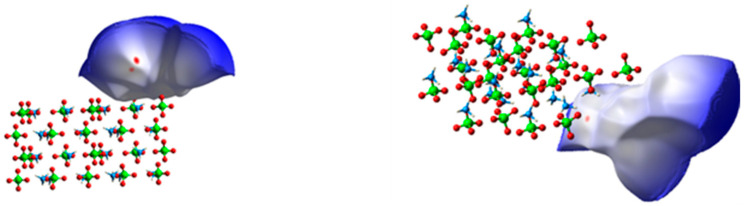
Hirshfeld surface (HS) generated with the PBT unit as the framework.

**Figure 7:**
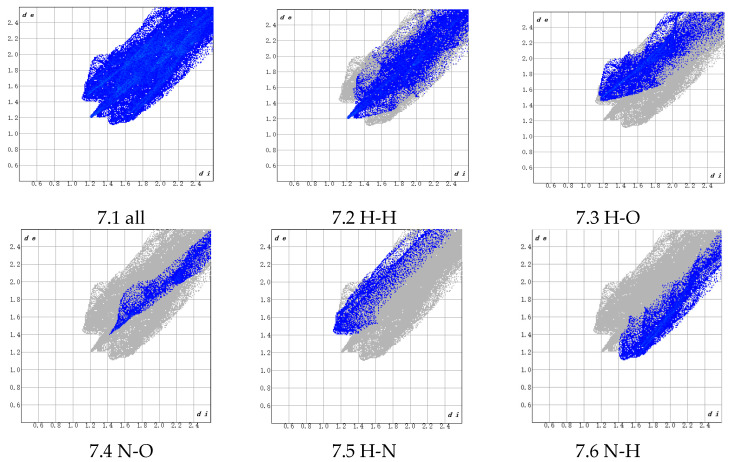
Fingerprint plot corresponding to the Hirshfeld surface (HS).

Next, Crystal Explorer (CE) [33] was utilized to analyze configuration (a). A single PBT unit adsorbed on the AP crystal surface was selected as the framework, and the Hirshfeld surface (HS) encompassing the framework atoms was calculated. Notably, while periodic boundary conditions were applied in prior simulations, they were intentionally removed in this HS calculation to isolate the target PBT unit and eliminate potential interference from neighboring periodic images. This approach ensures accuracy in analyzing localized interactions. The resulting HS and corresponding fingerprint plots are presented in Figure 6 and Figure 7, respectively.

As shown in Figure 6, the distance between the PBT unit and the AP crystal is relatively large. Atomic interactions can be clearly observed in Figure 7, where di denotes the distance from internal atoms to the HS, and de represents the distance from external atoms to the HS (unit: Å).

An analysis of Figure 7 reveals that the adhesion between the PBT unit and the AP crystal primarily arises from two types of interactions:

(1) H (PBT)–H (AP) interactions (45.3% contribution), which are predominantly driven by van der Waals forces and belong to non-bonded weak interactions. Although individual H-H interactions are weak, their high proportion suggests extensive van der Waals contacts between H atoms in the NH_4_^+^ groups on the AP crystal surface and H atoms in the THF chain segments (-CH_2_-CH_2_-O-) of PBT within the interfacial region. These interactions are enhanced by the cumulative effect of their abundance. The THF chain’s flexible structure further enables conformational adjustments to maximize the contact area, thereby improving adhesion.
(2) N (PBT)–H (AP) interactions (20.8% contribution), whose physical nature involves dipole–dipole interactions or weak hydrogen bonding. These originate from the partial negative charge (δ^−^) on the electronegative N atoms in PBT’s azide groups (-N_3_) interacting with the partially positive H atoms (δ⁺) in the NH_4_^+^ groups on the AP surface. These interactions, stronger than pure van der Waals forces, provide additional binding energy to the interface, enhancing adhesion stability.

Secondary interactions include the following: H (PBT)–O (AP) (11.7%), arising from dipole interactions between O atoms in AP’s ClO_4_^−^ and H atoms in PBT’s CH_2_ groups; H (PBT)–N (AP) (10.1%), originating from dipole interactions between N atoms in AP’s surface NH_4_^+^ and H atoms in PBT’s CH_2_ groups; N (PBT)–O (AP) (6.3%), resulting from interactions between N atoms in the azide groups and O atoms in AP’s ClO_4_^−^.

These findings align with the earlier charge density analysis, elucidating the forms and relative contributions of these weak interactions at the interface.

### 3.2. Molecular Dynamics Simulations

#### 3.2.1. Analysis of Simulated Annealing Results

After identifying the crystal surface with the lowest energy, we further determined the binding site with the lowest binding energy. Subsequent simulated annealing calculations were performed using VASP based on this optimized binding site structure. Simulations were conducted at 200 °C, 300 °C, 400 °C, and 1000 °C. The results revealed that during the heating/cooling cycles at 200 °C, neither the ionic structure of AP nor the atomic positions within the PBT unit exhibited significant displacement, thus requiring no further analysis. The simulated annealing results of 1000 °C demonstrated the complete disintegration of the system, indicative of a violent chemical reaction (i.e., detonation processes), which was also excluded from further analysis.

**Figure 8:**
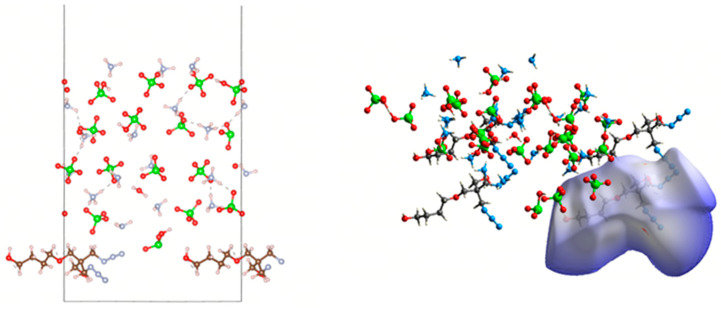
Structure from simulated annealing calculations at 300 °C.

**Figure 9:**
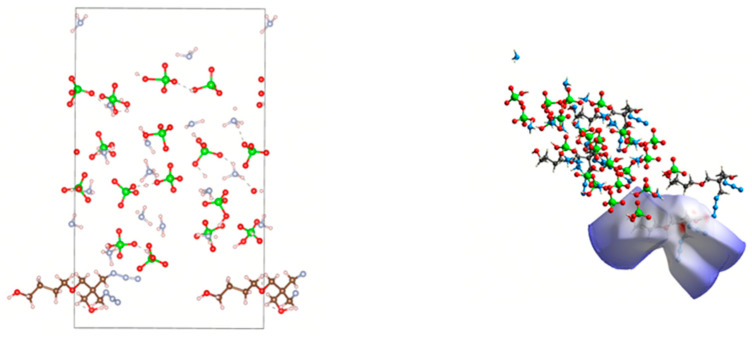
Structure from simulated annealing calculations at 400°C.

From the 300 °C (Figure 8) and 400 °C (Figure 9) simulation results, the following observations can be made: structural transformation: significant alterations occurred in the AP crystal structure during both annealing processes. A portion of hydrogen ions entered a dispersed state, suggesting that the AP crystal underwent localized liquefaction followed by re-solidification, ultimately forming a more stable composite structure with the PBT unit. Bond rearrangement: the pronounced cleavage of existing bonds and the formation of new bonds were observed within the AP crystal. Notably, hydrogen ions exhibited substantial displacement, indicating dynamic atomic reorganization during annealing.

**Figure 10:**
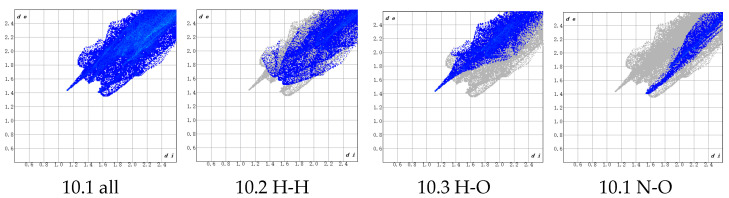
Fingerprint plot corresponding to the structure from simulated annealing at 300 °C.

As evident from Figure 8 (300 °C) and Figure 9 (400 °C), the Hirshfeld surfaces (HSs) demonstrate significantly stronger interactions between the PBT unit and ions in the AP crystal compared to the HSs in Figure 6, with shorter atomic distances. However, these interactions remain predominantly intermolecular forces, with no formation of true ionic bonds or hydrogen bonds. A further analysis of the 300 °C fingerprint plot (Figure 10) reveals the following: H (internal)–H (external) interactions: 34.5% of total bonds. H (internal)–O (external) interactions: 17.9%, characterized by a distinct peak, indicating strong localized interactions. N (internal)–O (external) interactions: 7.4%, representing weak contributions. Other atomic interactions were negligible.

**Figure 11:**
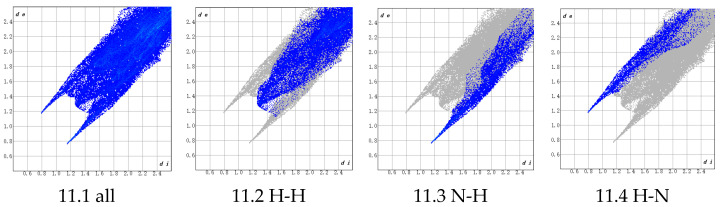
Fingerprint plot corresponding to the structure from simulated annealing at 400 °C.

In the fingerprint plot corresponding to the simulated annealing results at 400 °C (Figure 11), the H–H bonds account for a relatively large proportion of 46.4%. However, due to the longer atomic distances, the interactions are weak and can only be attributed to intermolecular forces. Although the bonding proportion between internal N atoms and external H atoms is only 10.8%, the significantly shorter atomic distances indicate stronger interactions. Additionally, the bonding proportion between internal H atoms and external N atoms is 4.7%, which is also low. However, the atomic distances are relatively close, with some H–N distances below 2 Å. Given that the typical range for hydrogen bonds is 1.5–3.0 Å, no chemical reactions occur in this configuration at 400 °C, and the hydrogen bonds remain the primary contributors to the adsorption interactions.

A comparison of Figure 10 and Figure 11 reveals that within the 300 °C range, an increase in temperature leads to enhanced ion activity in the AP crystal, ultimately resulting in tighter binding between the AP crystal atoms and the PBT unit. This process reduces the total system energy and increases the binding energy. However, under 400 °C conditions, the AP crystal structure undergoes significant changes, indicating the dissolution and re-solidification of the crystal lattice. Therefore, in subsequent molecular dynamics simulations, temperatures were maintained below 400 °C to ensure structural stability and prevent violent chemical transformations.

#### 3.2.2. Analysis of Mechanical Properties

We have elucidated the microscopic interaction mechanisms between the PBT unit and AP crystal, as well as the stable temperature range of the adsorption configurations. Therefore, we used molecular dynamics simulation to calculate the mechanical properties of the layered structure in Figure 4c under four temperature conditions, 238 K (−40 °C), 298 K (20 °C), 348 K (60 °C), and 498 K (120 °C), and performed tensile simulations to evaluate the mechanical performance of the PBT-AP layered structure at these temperatures.

The results show that at −40 °C, the material exhibits a bulk modulus of K = 0.355 GPa (low compressibility), a shear modulus of G = 0.4234 GPa, and a Young’s modulus (E) in the XYZ directions: X = 0.852 GPa, Y = 1.056 GPa, and Z = 0.700 GPa, indicating that the structure is stiffest along the Y-axis and softest along the Z-axis.

At 20 °C, the bulk modulus decreases to K = 0.240 GPa (increased compressibility), the shear modulus is G = 0.304 GPa, and the Young’s modulus (E) in the XYZ directions becomes X = 0.803 GPa, Y = 0.840 GPa, and Z = 0.596 GPa, confirming that the Z direction remains the softest under ambient conditions.

At 60 °C, the bulk modulus further decreases to K = 0.188 GPa (high compressibility), the shear modulus is G = 0.129 GPa, and the Young’s modulus (E) in the XYZ directions is X = 0.674 GPa, Y = 0.529 GPa, and Z = 0.427 GPa, showing that the structure becomes stiffest along the X-axis and softest along the Z-axis.

At 120 °C, the bulk modulus drops sharply to K = 0.066 GPa (extremely high compressibility), the shear modulus is G = 0.096 GPa, and the Young’s modulus (E) in the XYZ directions is X = 0.221 GPa, Y = 0.388 GPa, and Z = 0.310 GPa, indicating that the structure is stiffest along the Y-axis and softest along the X-axis. The X direction modulus decreases significantly, and the material’s anisotropy weakens substantially.

**Figure 12:**
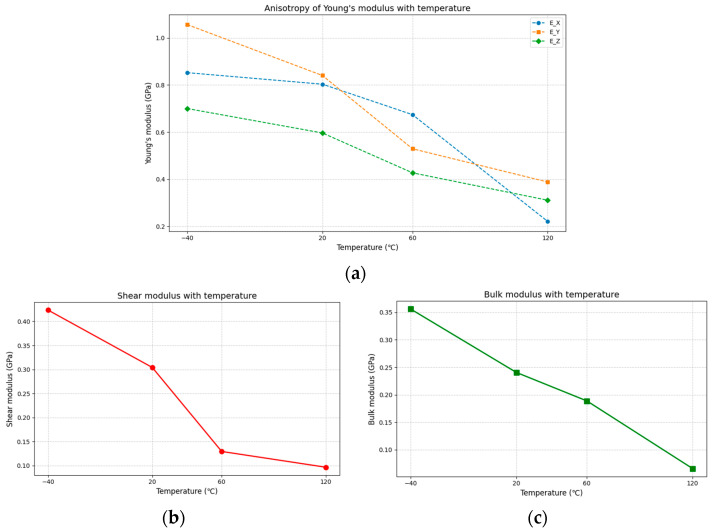
(**a**) Young’s modulus with temperature; (**b**) shear modulus with temperature; (**c**) bulk modulus with temperature.

The modulus variations across temperatures are shown in Figure 12. Figure 12a demonstrates a clear decreasing trend in Young’s modulus along the X, Y, and Z directions as the temperature increases. At low and ambient temperatures, the Z direction consistently exhibits the lowest Young’s modulus, with similar trends observed for the shear and bulk moduli, confirming that the material softens progressively with a rising temperature.

Overall, the structure maintains good compressibility, which enables effective cushioning in PBT-based solid rocket propellants. However, at elevated temperatures (especially above 60 °C), the viscosity of the PBT binder decreases markedly, reducing its adhesive performance.

#### 3.2.3. Tensile Simulation Results

**Figure 13:**
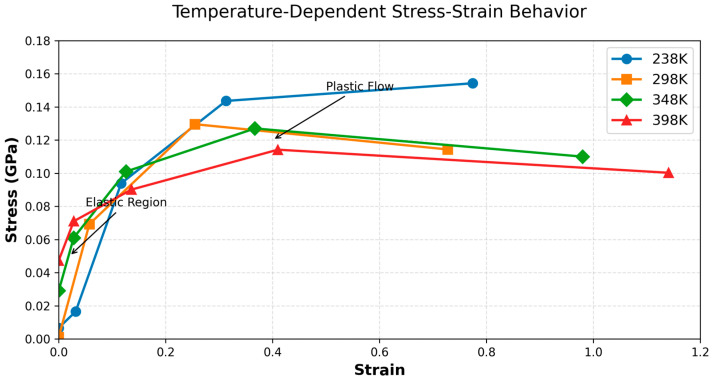
Stress–strain curves under different temperatures.

The stress–strain curves from the tensile simulations are shown in Figure 13. The curves for all four temperatures exhibit similar trends: in the strain range of 0 to 0.4, the stress increases, indicating that the model maintains good elasticity at small deformations. Beyond a strain of 0.4, only the −40 °C curve continues to show increasing stress, while stresses at other temperatures decline, signifying the onset of plastic deformation. The input stresses during simulation were 0, 0.5, 1, 2, and 3 GPa. However, the material’s actual stress response was significantly lower than the input stresses, with maximum strain values exceeding 1.0, demonstrating the material’s intrinsic stretchability. To preserve elasticity and avoid fracture, strains should be limited to below 0.3. A comparison of the stress–strain curves across temperatures reveals that before plastic deformation, the behaviors at −40 °C and 20 °C are nearly identical. This indicates that the PBT binder retains consistent mechanical properties from low to ambient temperatures, ensuring reliable adhesion performance. The slopes of the curves in the strain range exceeding 0.4 at 20 °C and 60 °C show almost no discernible difference. This phenomenon may be attributed to the glass transition temperature (T_g_ ≈ 34 °C) of the PBT material, which can also explain why the maximum elongation at 60 °C is significantly higher than that at 20 °C. Notably, at larger strains, the binder exhibits enhanced viscosity under low-temperature conditions.

## 4. Conclusions

In this study, DFT calculations and MD simulations were performed, identifying four relatively stable adsorption configurations on the (001) crystal surface. The calculated adsorption energies for these configurations were −10.039 eV, −9.943 eV, −9.981 eV, and −9.925 eV. A subsequent charge density analysis of the most stable configuration revealed the interaction mechanism between the PBT unit and AP crystal in the PBT-based insensitive propellant: the interactions are dominated by intermolecular van der Waals forces, and the binding effects of PBT on AP are evenly distributed across the crystal surface. We conclude that this homogenization of electron transfer enables stable adsorption between PBT and AP while minimizing chemical reactivity, thereby endowing the structure with enhanced chemical and physical stability. From the Hirshfeld surfaces (HSs) and fingerprint plots, it is evident that 66% of the total bonds involve interactions between H and N atoms in the PBT unit and H atoms in AP. This conclusively demonstrates that N and H atoms are the primary functional contributors within the PBT unit.

The simulated annealing results demonstrate that increasing temperature significantly enhances the binding strength between the PBT unit and AP crystal. Furthermore, at elevated temperatures, interactions between H atoms within the PBT unit and external atoms become more pronounced, with marked reductions in distances for H–H, H–N, and N–H interactions reaching peak proximity values of 1.2 Å (internal) and 0.8 Å (external). Additionally, the total energy of the system decreases substantially after structural relaxation.

Molecular dynamics simulations were conducted to evaluate the mechanical properties of the PBT-AP interface model and its stress–strain behavior during tensile deformation at four temperatures: 238 K (−40 °C), 298 K (20 °C), 348 K (60 °C), and 498 K (120 °C). An analysis of the results indicates that the PBT binder retains excellent adhesion performance at low temperatures but exhibits significant viscosity reduction at elevated temperatures (particularly above 60 °C). These findings provide critical guidance for the design and optimization of PBT-based solid propellants.

The PBT material, which in reality is a highly polymerized chain-like structure, was simplified to a monomeric unit in this study to ensure computational feasibility for DFT and limited-range MD simulations. Despite this simplification, this work represents the first atomic-level investigation of the interaction mechanisms and strength at the PBT-AP interface. The results hold significant reference value for improving existing PBT solid rocket propellant formulations. By leveraging molecular dynamics simulations, theoretical adjustments to formulations can be prioritized before experimental validation, substantially reducing development costs and time-to-market. This approach also enables the tailored design of materials to meet specific performance requirements with greater efficiency.

## Figures and Tables

**Table 1:** PBT unit, AP crystal, PBT-AP system, and adsorption energy (eV).

	E_suf_ (eV)	E_clean_ (eV)	E_total_ (eV)	E_ab_ (eV)
a	−212.917	−748.900	−971.857	−10.039
b	−212.917	−748.900	−971.758	−9.940
c	−212.917	−748.900	−971.628	−9.810
d	−212.917	−748.900	−971.743	−9.925

## Data Availability

The data presented in this study are available on request from the corresponding author. The data are not publicly available due to research privacy.

## Abbreviations

MD	molecular dynamics
DFT	Density functional theory
AP	ammonium perchlorate
PBT	poly(3,3-bis-azidomethyl oxetane)-tetrahydrofuran
vdW	van der Waals
CTPB	carboxyl-terminated polybutadiene
HTPB	hydroxyl-terminated polybutadiene
PAW	Projected Augmented Wave
PBE	Perdew–Burke–Ernzerhof
GGA	generalized gradient approximation
